# *Predictors of microalbuminuria and its relationship with glycemic control among* Type 2 diabetic patients of Jazan Armed Forces Hospital, southwestern Saudi Arabia

**DOI:** 10.1186/s12902-022-01232-y

**Published:** 2022-12-08

**Authors:** Hassan Ali Abdelwahid, Hesham Mohamed Dahlan, Gassem Maoudhah Mojemamy, Gasem Hussein Darraj

**Affiliations:** 1 Jazan Armed Forces Hospital (JAFH), Suez Canal University, Ismailia, Egypt; 2 JAFH, Jazan, Saudi Arabia

**Keywords:** Microalbuminuria, Macroalbuminuria, Type 2 diabetes mellitus, Glycemic control

## Abstract

**Background and purpose:**

Diabetic kidney disease (DKD) is highly prevalent among patients with diabetes mellitus. It affects approximately 20% of diabetic patients, who are believed to be more than 400 million individuals. The objectives of the present work were to assess patterns of albuminuria and determine microalbuminuria predictors among patients living with type 2 diabetes (T2D) who attended the family medicine department of Jazan Armed Forces Hospital.

**Methods:**

A case–control design was used and included two groups (n, 202/group), one with microalbuminuria and the other with a normal urine albumin/creatinine ratio (ACR). Data regarding patient history, glycosylated hemoglobin (HbA1c), lipid profile, renal function tests, ACR, ASCVD (atherosclerotic cardiovascular disease) risk, etc., were collected.

**Results:**

The prevalence rates of microalbuminuria and macroalbuminuria were 26.4% and 3.9%, respectively. HbA1c was significantly higher in patients with microalbuminuria (9.3 ± 2.2; P˂0.001) and macroalbuminuria (10.5 ± 2.3; P˂0.001) than in those with normal ACR (8.3 ± 1.9%). The predictors of microalbuminuria were poor glycemic control with HbA1c ≥ 7% {OR, 2.5 (95% C. I, 1.5–4.2)}; hypertension {(OR, 1.8 (95% C. I, 1.2–2.8)}; estimated glomerular filtration rate (eGFR) of ˂90 mL/min/1.73 m2 {OR, 2.2 (95% C. I, 1.4–3.6}; smoking {OR, 1.3 (95% C. I, 0.7–2.6}; and body mass index {OR, 1.05 (95% C. I, 1.01–1.09}.

**Conclusion:**

Microalbuminuria is highly prevalent among patients with type 2 diabetes and is associated with poor glycemic control and hypertension, necessitating aggressive and timely screening and treatment.

## Introduction

The early clinical manifestation of diabetic nephropathy is an increase in urinary protein excretion, and DKD is defined as albuminuria plus or minus a progressive decrease in eGFR in the setting of long-standing diabetes (> 10 years' duration of type 1 diabetes and at the time of diagnosis of type 2 diabetes). It may be associated with retinopathy [1].

Albuminuria is classified into three grades (A1, A2, and A3) by the albumin creatinine ratio (ACR) in a spot urine sample. The first grade is known as normal to mildly increased albuminuria, in which ACR is < 30 mg/g. The second grade, A2, is moderately increased albuminuria with an ACR of 30–300 mg/g (the new terminology for what was formerly called "microalbuminuria") [2]. The 3rd grade, A3, is severely increased albuminuria (previously known as macroalbuminuria, or dipstick positive proteinuria) with an ACR of > 300 mg/g or AER (albumin excretion ratio) of > 300 mg/24 h. The risk of developing overt DKD is associated with the rates of albumin excretion. It should be noted that albuminuria is an independent definition of chronic kidney disease (CKD) even if GFR is > 60 mL/min/1.73 m^2^ [3].

Approximately 20% to 40% of patients living with type 1 or type 2 diabetes mellitus will develop DKD [1, 4]. The epidemiology of DKD has been best studied in patients with type 1 diabetes (T1D) since the time of clinical onset is usually known. Worldwide, diabetes mellitus is the most common cause of chronic kidney disease (CKD), and approximately 20% to 30% will have microalbuminuria after a mean duration of diabetes mellitus of 15 years, but it may be present at the time of diagnosis of type 2 diabetes [1, 5]. The DKD prevalence is increasing and reflects the rise in the prevalence of diabetes worldwide [4, 6]. Although in the past it was stated that the risk of nephropathy was lower in T2D than in T1D [7], the literature revealed that albuminuria progression was more common in T2D versus T1D [8, 9].

In the Kingdom of Saudi Arabia, the prevalence of diabetic nephropathy was 10.8% (1.2% microalbuminuria, 8.1% macroalbuminuria, and 1.5% end-stage renal disease, ESRD) in a nationwide study that included a large number of patients living with T2D (n, 54,670) in 2013 [10]. However, higher prevalence figures for albuminuria were reported in other studies. For example, DKD affected 18.9% (15.2 with microalbuminuria and 3.7% with macroalbuminuria) in the diabetic center of Prince Mansour Military Hospital of Taif City [11]; 33.2% in primary healthcare clinics of King Fahad Armed Forces Hospital, Jeddah; and 54.3% in primary health care of Abha City (54.3%) [12].

DKD screening by early detection of microalbuminuria is reported to be cost-effective in patients with diabetes and hypertension [13]. Patients living with T2D should be screened by the albumin to creatinine ratio (ACR) at the time of diagnosis of diabetes and annually in patients with type 1 diabetes with a duration of ≥ 5 years and in all patients with type 2 diabetes with comorbid hypertension [13, 14].

The pattern of albuminuria in Jazan Armed Forces Hospital (JAFH) is unknown, therefore a study for the early detection and management of microalbuminuria is urgently needed. The objectives of the present work were to assess patterns of albuminuria and determine microalbuminuria predictors among patients living with T2D who attended the family medicine (FM) department of Jazan Armed Forces Hospital.

## Materials and Methods

### Study Area and study population

The present study was performed at the family medicine department, Jazan Armed Forces Hospital (JAFH), Saudi Arabia. The target population included all patients eligible for medical care in JAFH (approximately 100,000), and the study population consisted of patients living with T2D who attended FM clinics that are affiliated with JAFH.

The electronic medical records and computerized CIC (chronic illness clinic) data were used to select patients who met the inclusion criteria, as well as to assess the relevant history and laboratory workup of each identified patient. The CIC was constructed in JAFH in October 2018 to provide integrated and evidence-based multidisciplinary care. The CIC scope of services included evaluation of newly diagnosed diabetic patients, screening, and prevention of complications, providing health education about diabetes and lifestyle modification, delivering advanced antihyperglycemic pharmacotherapy, and referral to specialist care if needed.

### Study design

A case–control design was used in the present work and included two groups, one with the outcome of interest (microalbuminuria) and the other with normal ACR.

### Inclusion criteria

1. Patients living with T2D.
2. Age ≥ 18 years.
3. Eligible for medical care at JAFH.
4. Urine ACR during the period from January 2020 to January 2021.
5. Conducted HbA1c and other annual diabetes Lab panels during 2019.

### Exclusion criteria

1. Patients living with T1D.
2. Female patients with gestational diabetes.
3. Patients with urinary tract infections.
4. Patients with other conditions that could alter albuminuria (e.g., autoimmune diseases such as IgA nephropathy and glomerulonephritis, polycystic kidney disease, etc.).

### Sample design

The sample size was calculated first using an appropriate equation to justify the study sample and then selected from the registered CIC patients' data. The sample size of the case–control design was 202/group based on expected exposure in the controls to be 0.32 and assumed odds ratio of 1.8 (from a previous study) [15] with a total sample of 404 in both groups.

Patients living with T2DM and microalbuminuria (n,202) were included in the case–control analysis with a similar number in the control group (n, 202) who were selected randomly by a simple random sampling method from those with normal ACR (n, 532) using random number tables. Patients with macroalbuminuria were excluded from the case–control analysis.

### Data collection

Data regarding the outcome variables were collected from the CIC electronic medical records. The pooled cohort equation [16] was used according to the CIC policy to calculate the ASCVD risk that was collected from the patient's file. Data regarding HbA1c, lipid profile, renal function tests (serum urea and creatinine), urine analysis to exclude urinary tract infection (UTI), and urine albumin/creatinine ratio were collected. The urine ACR was assessed by the quantitative method using a morning spot urine specimen to avoid the effect of orthostatic proteinuria. A COBAS E 501 analyzer was used to assay urine albumin and creatinine with a calculation of albumin/creatinine ratio using CREJ2 and ALBT2 kits from Roche Diagnostics.

The data collection was started after we obtained ethical approval from the hospital Research and Ethics committee from March 2022 to the end of April 2022, and the study was completed in May 2022. The study was conducted in accordance with Helsinki Declaration, following research ethical guidelines [17].

### Statistical design

The Statistical Package for Social Sciences (SPSS version 16.0) was used for data analysis. Continuous variables were compared using one-way analysis of variance (ANOVA), and the chi-square test was conducted for categorical variables to assess the significance of differences in baseline characteristics of the three grades of albuminuria. Multinomial logistic regression with a main effects model was conducted to identify the significant predictors of microalbuminuria. Patients with macroalbuminuria were not included in the multinomial logistic regression. The chi-square of likelihood ratio tests for model fitting criteria was assessed to test the overall fit of the model. p < 0.05 was considered the significance cutoff point.

## Results

**Fig. 1:**
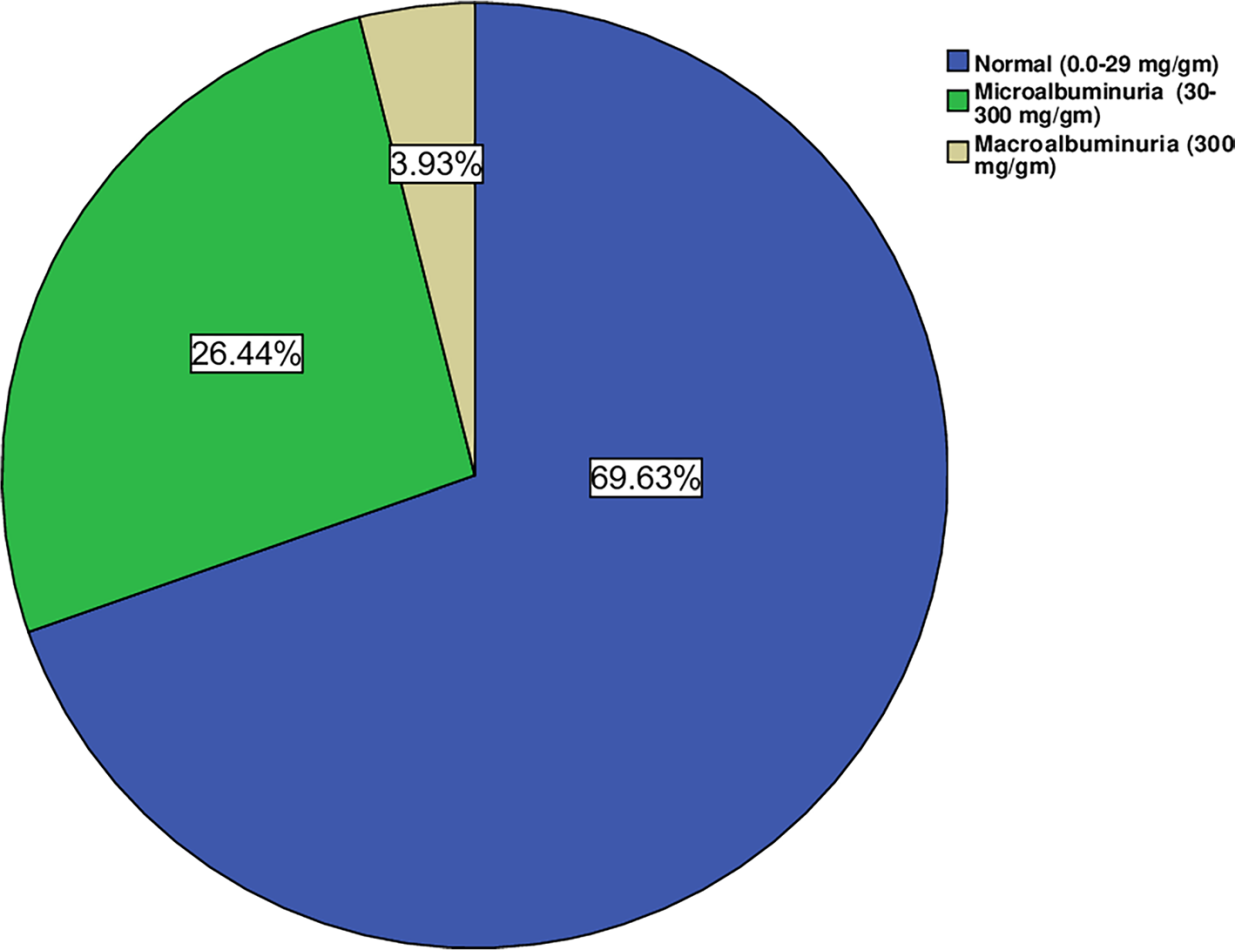
Patterns of albuminuria among the study group (n, 764)

In 2021, the CIC records of patients living with T2D included 764 who met the inclusion criteria. The total number of males was 344 (45.0%) and that of females was 420 (55.0%). Their ages ranged from 22 to 90 years, with a mean of 54.5 ± 12.6 years. The age of males, 54.3 ± 12.6, was not significantly different (t value, 0.398 and P, 0.691) from that of females, 54.7 ± 12.7 years. The study participants were classified into three categories or grades, A1, A2, and A3, based on the urine ACR. Category A1 included T2D with normal to mildly increased albuminuria (ACR < 30 mg/gm; n, 532). The second category, A2, included those with microalbuminuria with an ACR of 30–300 mg/gm; n, 202. The 3rd one, A3, was severely increased albuminuria with an ACR of > 300 mg/gm; n, 30. There were no significant differences between A1 (n, 532) and A2 (n, 202) regarding patients' age, sex, and duration of diabetes (not illustrated in the tables). The prevalence rates of microalbuminuria and macroalbuminuria were 26.4% (202/764) and 3.9% (30/764), respectively, as illustrated in Fig. 1, with no end-stage renal disease (ESRD).

One-way analysis of variance (ANOVA) was conducted to test the significance of differences between the three categories of urine albumin creatinine ratio (ACR) regarding continuous variables. The Scheffe multiple comparison test was used to identify the significantly different groups. The patients with microalbuminuria and macroalbuminuria had significantly (P˂0.05) higher levels of HbA1c than those with normal ACR (control group). The body mass index (BMI) of patients with microalbuminuria was significantly higher than that of the control group. Additionally, the eGFR (estimated glomerular filtration rate) of diabetic patients with micro- or macroalbuminuria was significantly lower than that of the control group (P˂0.05). On the other hand, there were no significant differences between the three groups regarding patient age, duration of diabetes, or lipid profile, as illustrated in Table 1.

**Table 1:** Baseline characteristics of the study participants with and without albuminuria*

	Control group^₲^	Microalbuminuria cases ^▲▲^	Macroalbuminuria cases**
**Age in years**	53.5 (12.3)	55.1 (13.6)	54.7 (13.8)
**Duration of diabetes (years)**	7.6 (7.2)	7.7 (7.1)	6.4 (6.2)
**HbA1c (%)**	8.4 (1.9) ^▲^	9.3 (2.2) ^▲, ♣^	10.5 (2.3) ^▲, ♣^
**TC (mg/dL)**	183.0 (40.3)	183.7 (44.2)	191.2 (45.0)
**LDL (mg/dL)**	114.0 (38.5)	115.5 (43.2)	121.0 (46.4)
**HDL (mg/dL)**	45.3 (11.5)	46.4 (12.9)	47.8 (13.5)
**Tg (mg/dL)**	144.9 (74.3)	136.6 (64.5)	139.1 (74.1)
**eGFR (mL/min/1.73)**	102.2 (21.3) ^▲^	94.1 (22.9) ^▲,♣^	78.9 (30.4) ^▲, ♣^
**BMI (kg/m**^**2**^**)®®**	30.6 (5.9)	32.4® (6.7)	27.8 (2.1)

^₲^, Control group with normal urine ACR, n, 202; ^▲▲^, Microalbuminuria (cases, n, 202) with ACR: 30–300;**, Macroalbuminuria, n,30, with ACR˃300;*, mean and standard deviation in parentheses; ^▲^, The control group is significantly different from the other two groups; ^♣^, microalbuminuria is significantly different from the macroalbuminuria group; ®, Microalbuminuria is significantly different from the control and macroalbuminuria groups; HbA1c, glycosylated hemoglobin; TC, total cholesterol; LDL, low-density lipoprotein; HDL, high-density lipoprotein; TG, triglycerides; and eGFR, estimated glomerular filtration rate; ®®, body mass index in ^kg/m2x^

The three grades of urine ACR were cross-tabulated with some possible categorical predictors. There were significant differences between the microalbuminuria group and the control group regarding hypertension and HbA1c. The prevalence of controlled diabetes with HbA1c ˂7% was significantly higher in the control group with normal ACR (30%) compared to 17% and 7% in the microalbuminuria and macroalbuminuria categories, respectively (Table 2).

**Table 2:** The relationship between albuminuria and its possible predictors by bivariate analysis*

	Control group^₲^	Microalbuminuria (cases)^▲▲^	Macro**
**Sex**			
Males Females	93 (46.0) 109 (54.0)	83 (43.1) 119.0(58.9%)	6 (20) ^♥♥^ 24 (80)
**Hypertension**			
Present Absent	119 (58.9) 83 (41.1)	139 (68.8) ^♥♥^ 63 (31.2)	23 (76.7) 07 (23.3)
**eGFR**			
≥ 90 mL/min/1.73 m^2^ <90 mL/min/1.73 m^2^	143 (70.8) 59 (29.2)	125 (61.9) 77 (38.1)	16 (53.3) 14 (46.7)
**High density lipoproteins (mg/dL)**^♣^			
In target Above the target	96 (47.5) 106 (52.5)	101 (50.0) 101 (50.0)	15 (50) 15 (50)
**Triglycerides**^▲^			
In the target (˂150 mg/dL) Above target (≥ 150 mg/dL)	145 (71.8) 57 (28.2)	144 (71.3) 58(28.7)	21 (70) 9 (30)
**Low density lipoproteins**			
In the target (≥ 100 mg/dL) Above target (˂100 mg/dL)	83 (41.1) 119 (58.9)	86 (42.6) 116 (57.4)	12 (40) 18 (60)
**ASCVID**^♥^			
Less Than 10%	95 (47.0)	107 (53.0)	7 (23.3) ^♥♥^
More than or equal 10%	107 (53.0)	95 (47.0)	23 (76.7)
**HbA1C**			
Less than 7 gm % ≥ 7 gm %	60 (29.7) 142 (70.3)	35 (17.3) ^♥♥^ 167 (82.7)	2 (6.7) ^♥♥^ 28 (93.3)
**Smoking**			
smoker or ex-smoker Non Smoker	18(8.9) 184 (91.1)	26 (12.9) 176 (87.1)	4 (13.3) 26 (86.7)

^₲^, Control group with normal urine ACR, n, 202; ^▲▲^, Microalbuminuria (cases, n, 202); **, Macroalbuminuria, n,30; *, number and percent in parenthesis; ^♣^ At the target level (more than 40 in males and 50 in females) and not at the target level (less than 40 in males and 50 in females); ^♥♥^, significantly different from diabetic patients with normal urine ACR (P value of Chi-Square ˂0.05); ^♥^, atherosclerotic cardiovascular disease risk

Multinomial logistic regression with a main effects model was conducted to identify the significant predictors of microalbuminuria. The dependent variable involved patients with microalbuminuria (n, 202) versus the control group with normal ACR (n, 202) as a reference group. The independent variables of the multinomial logistic regression included factors (categorical variables that are listed in Table 2) and covariates (age, duration of diabetes, and BMI as continuous variables). The main-effects model contained all covariate and factor main effects and was fitted in a multivariable model at once. The chi-square of likelihood ratio tests for model fitting criteria was highly significant, P˂ 0.001. The results showed that the significant predictors of microalbuminuria were poor glycemic control, hypertension, smoking, and eGFR ˂ 90 mL/min/1.73 m2 with ORs of 2.5, 1.8, 1.3, and 2.2, respectively. BMI had a significant but small OR of 1.02 (Table 3).

**Table 3:** Predictors of microalbuminuria among the study group*

	B-coefficient	Wald Chi-square	P value	Odds Ratio	95.0% C.I. of Odds Ratio
Duration of Diabetes in years^©^	-0.006	0.135	0.713	0.994	0.964–1.026
Age in years^©^	0.015	2.954	0.086	1.015	0.998–1.033
Body mass index^©^	0.047	7.605	**0.006**	1.049	1.014–1.085
Hypertension	0.589	7.087	**0.008**	1.803	1.168–2.783
Sex	0.244	1.159	0.282	0.780	0.497–1.226
ASCVD^♥^ (≥ 10)	0.300	1.715	0.190	0.741	0.472–1.161
Uncontrolled HbA1c (≥ 7%)	0.935	12.918	**0.000**	2.547	1.530–4.241
eGFR (˂90 mL/min/1.73m^2^)	0.808	10.753	**0.001**	2.242	1.384–3.634
LDL in the target♥♥	0.029	0.018	0.892	1.029	0.676–1.568
HDL in the target^♣^	0.017	0.006	0.936	1.017	0.670–1.545
TG in the target^▲^	-0.158	0.432	0.511	0.854	0.533–1.369
Smoking	0.264	0.567	**0.451**	1.302	0.655–2.587

^*^ The reference group included the control group with normal ACR;^©^, continuous variables as covariates; ^♥^, atherosclerotic cardiovascular disease risk; ♥♥, low-density lipoprotein in the target (˂150 mg/dL);^♣^, high-density lipoprotein in the target level (more than 40 in males and 50 in females) and not in the target level (less than 40 in males and 50 in females);▲, triglycerides in the target (˂150 mg/dL)

## Discussion

The prevalence of microalbuminuria and macroalbuminuria in the current study was 26.4 and 3.9, respectively, a result that is consistent with that of a cross-sectional study conducted in Pakistan on 133 patients living with type 2 diabetes, with reported prevalence rates of 25.6% and 4.5% for microalbuminuria and macroalbuminuria, respectively [18]. However, there is a wide discrepancy in the DKD prevalence figures at the international level, with a prevalence rate ranging from 20 to 40% [1, 4]. The kingdom of Saudi Arabia is not excused from this wide variation. For example, DKD prevalence was 10.8% (1.2% microalbuminuria, 8.1% macroalbuminuria, and 1.5% ESRD) in a nationwide study that included a large number of patients living with T2D (n, 54,670) who were selected from the Saudi National Diabetes Registry [10]. In another study conducted in the diabetic center of Prince Mansour Military Hospital of Taif city, the prevalence of DKD was 18.9% (15.2 with microalbuminuria and 3.7% macroalbuminuria) [11]. On the other hand, higher prevalence figures for microalbuminuria were detected in the Primary Health Care Clinics of King Fahad Armed Forces Hospital, Jeddah (33.2%), and a primary health care center in Abha City (54.3%) [12]. The inconsistency in the reported prevalence figures of microalbuminuria at the national and international levels may be attributed to variations in genetic susceptibility to nephropathy in different study populations, methodologies of different studies, and patient characteristics that may lead to variations in the risk factors for DKD. The urine ACR quantitative method used in the current study is a more accurate way to assess albuminuria than semiquantitative dipstick tests, which might be used in other studies for microalbuminuria screening.

In the present study, glycosylated hemoglobin (HbA1c) was significantly higher in diabetic patients with microalbuminuria (9.3 ± 2.2) and macroalbuminuria (10.5 ± 2.3) than in those with normal ACR (8.3 ± 1.9%). This means that an increase in HbA1c levels was associated with an increase in urine ACR, a result that is consistent with other studies [11, 12, 17]. It was reported that the risk for microalbuminuria increases significantly at HbA1c levels ≥ 5.5% [19]. There are no large trials that have studied appropriate glycemic targets to prevent DKD. The American Diabetic Association recommends a target A1C level of less than 7% for many adults. A lower HbA1c target (e.g., less than 6% vs. 7% to 8%) has been associated with a decrease in DKD but at the cost of more hypoglycemia, polypharmacy, and increased mortality [20].

The results of the present work showed that the odds ratios (ORs) of poor glycemic control (HbA1c ≥ 7%) and being hypertensive were 2.5 and 1.8, respectively, in type 2 diabetic patients with microalbuminuria (MA). In patients with T2DM, observational studies have reported that poor glycemic control is associated with the development of MA and good glycemic control has been shown to prevent the development of DKD and to regress the established pathology [21].

In the present study, the association of T2D and essential hypertension increased the prevalence rate of microalbuminuria from 31.2 to 68.5%. Similar results were reported in a microalbuminuria prevalence study that was conducted to assess the prevalence of microalbuminuria among type 2 diabetic patients with hypertension in Asia [22]. Therefore, the presence of hypertension increases the risk of microalbuminuria, necessitating appropriate management of hypertension in addition to diabetes.

In a recent meta-analysis, the authors demonstrated that both eGFR and ACR independently as well as in combination provide statistically significant improvement in the prediction of CVD events [23]. In the present study, patients with microalbuminuria were two times more likely to have an eGFR ˂ 90 mL/min/1.73 m2 {OR, 2.2 (95% C. I, 1.4–3.6)}, a result that is consistent with another study that reported a positive correlation between the albumin excretion ratio and eGFR < 60 mL/min/1.73 m2 [23]. Therefore, we can conclude that these two parameters provide a complimentary benefit in the management of cases with CKD and diabetes [23–25].

In the present study, microalbuminuria was more prevalent in smokers who were 1.3 times more likely to have microalbuminuria {OR, 1.3 (95% C. I, 0.7–2.6)}. It was reported that diabetic smokers had higher prevalence rates of microalbuminuria in both T1D (18 vs. 14%) and T2D (20% vs. 13%), and it was independently associated with elevated HbA1c levels (p < 0.001) and microalbuminuria (p < 0.001) in both T1D and T2D [26].

Our findings indicate that BMI had a significant but small OR, 1.02, for microalbuminuria. Additionally, the BMI of microalbuminuria cases (32.4 ± 6.7 kg/m2) was significantly higher than that of the control group (30.6 ± 5.9 kg/m^2^). Obesity has been reported as an independent risk factor for CKD even in the absence of diabetes, and albuminuria has been shown to improve with weight loss in several series [27, 28]. Increased BMI has been linked to deterioration of renal function as well as increased risk of ESRD [26]. These findings encourage comprehensive and motivated weight loss.

The study has some limitations. For example, it is a retrospective and monocentric study that did not allow for assessment of albuminuria prognosis or to include other possible risk factors that may affect the results, such as lifestyle and eating habits.

## Conclusion

In summary, it can be concluded based on the results of the present work that microalbuminuria was highly prevalent among patients with type 2 diabetes. It was associated with poor glycemic control, eGFR decline, hypertension, smoking, and increased BMI, necessitating aggressive and timely screening and treatment. Family physicians are in the best position to provide screening for early detection and management of diabetes, hypertension, DKD, obesity, and smoking, as appropriate management of common health problems is one of the important objectives of primary health care for the individual and families in the community by using scientifically sound methods and technology according to Declaration of Alma-Ata, 1978 [29].

## Data Availability

Electronic medical records are classified as sensitive data in Jazan Armed Forces Hospital, and data sharing via public deposition is not allowed. The data will be available upon reasonable request to the corresponding author.

## Abbreviations

A1: Normal to mildly increased albuminuria, in which ACR is < 30 mg/gm
A2: Moderately increased albuminuria with ACR of 30–300 mg/g
A3: Severely increased albuminuria with an ACR of > 300 mg/g
ACR: Urine albumin/creatinine ratio
ANOVA: Analysis of variance
ASCVID: Atherosclerotic cardiovascular disease
BMI: Body mass index in kg/m^2^
C. I: Confidence interval
CIC: Chronic Illness Clinic
CKD: Chronic Kidney Disease
DKD: Diabetic Kidney Disease
eGFR: Estimated glomerular filtration rate
ESRD: End-Stage Renal Disease
FM: Family Medicine
HbA1C: Glycosylated hemoglobin
HDL: High-density lipoprotein
JAFH: Jazan Armed Forces Hospital
LDL: Low Density Lipoprotein
MA: Microalbuminuria
OR: Odds Ratio
T1D: Type 1 Diabetes
T2D: Type 2 diabetes
T2DM: Type 2 diabetes mellitus
TC: Total Cholesterol
TG: Triglycerides
